# Vascular patterns in reactive lymphoid tissue and in non-Hodgkin's lymphoma

**DOI:** 10.1038/sj.bjc.6600742

**Published:** 2003-02-18

**Authors:** E Passalidou, M Stewart, M Trivella, G Steers, G Pillai, A Dogan, I Leigh, C Hatton, A Harris, K Gatter, F Pezzella

**Affiliations:** 1 3rd Department of Respiratory Medicine, Sismanogleio Hospital, Sismanogleio 1, PC 15126 Athens, Greece; 2 Cancer Research UK Tumour Pathology Group, Nuffield Department of Clinical Laboratory Sciences, John Radcliffe Hospital, Headington, Oxford OX3 9DU, UK; 3 Department of Haematology, John Radcliffe Hospital, Headington, Oxford OX3 9DU, UK; 4 Cancer Research UK Medical Oncology Unit, Churchill Hospital, University of Oxford, Oxford; 5 Cancer Research UK Medical Statistics Group, Centre for Statistics in Medicine, Institute of Health Sciences, Oxford; 6 Department of Histopathology, University College London, London; 7 Centre for Cutaneous Research, Barts and the London, Queen Mary's School of Medicine and Dentistry, London

**Keywords:** lymphoma, lymph node, angiogenesis, vascular regression, vascular phenotype

## Abstract

The few studies published on angiogenesis in lymphoma have raised the question of whether or not microvessel density (MVD) is associated with more aggressive disease and have reported the observation that in follicular lymphomas, vessels are mature rather than immature. We investigated MVD and the vascular phenotype within follicular or diffuse large B-cell lymphomas, reactive nodes and tonsils. Vascular phenotype was defined by the expression or loss of reactivity to the antibody LH39 (detecting the LH39 laminin epitope of the basement membrane in mature vessels) and by detection of *α*V*β*3 (expressed on immature vessels). In reactive nodes and in follicular lymphomas, MVD was higher in the paracortex than in germinal centres or in neoplastic follicles. However, in neoplastic follicles an increase in *α*V*β*3-positive endothelium suggested the activation of an angiogenic pathway different from that present in the reactive follicles. In large B-cell lymphomas, MVD was higher than in reactive and neoplastic follicles but lower than in the reactive paracortex. The number of immature vessels (LH39 negative) and of *α*V*β*3-positive vessels was higher than in reactive lymph nodes and follicular lymphoma suggesting that a switch to a different angiogenic pathway has occurred. Finally, we have demonstrated that within reactive and neoplastic follicles vascular regression is occurring, perhaps constraining the growth of reactive follicles alongside other phenomena such as apoptosis. Vascular regression was previously believed to occur in adults only in ovarian and endometrial tissue. We conclude that different types of angiogenesis are present in follicular lymphomas and large B-cell lymphomas. This has implications for possible future therapies.

Evidence supporting a role for neoangiogenesis in haematological malignancies has been found in lymphoid (Perez-Atayde *et al*, 1997; Kini *et al*, 1998; Veiga *et al*, 1998) and myeloid leukaemias (Fiedler *et al*, 1997; Shami *et al*, 1998; Pruneri *et al*, 1999), myeloproliferative disorders, myelodysplasia (Aguayo *et al*, 1998; Pruneri *et al*, 1999) and myeloma (Vacca *et al*, 1994,1999a; Raza *et al*, 1999). In non-Hodgkin's lymphomas, expression of angiogenic factors in cell lines (Bellamy *et al*, 1999) and tissue (Doussis-Anagnostopoulou *et al*, 1997; Foss *et al*, 1997; Turley *et al*, 1998) has been demonstrated.

Although these studies suggest a role for angiogenesis in lymphomas several questions remain unanswered.

First, it is not clear whether high microvessel density (MVD) is associated with more aggressive lymphomas. A group of authors (Ribatti *et al*, 1996; Vacca *et al*, 1999b) reported that MVD is higher in lymphomas than in reactive nodes and is higher in aggressive than in indolent lymphomas. However, MVD in reactive nodes has been found to be higher (Ridell and Norrby, 2001) or comparable (Arias and Soares, 2000) to that observed in lymphomas, including large cell lymphomas.

A second intriguing point is the observation by Hyjek *et al* (Hyjek *et al*, 1999) that in follicular lymphomas, vessels have mostly a mature phenotype (i.e. have pericytes) rather than an immature as commonly reported in malignancies.

All these authors agree on two points: the neoplastic follicles in follicular lymphoma appear to have fewer vessels than the surrounding paracortex and the high-grade lymphomas. They also noticed that staining for vascular markers highlights a similar vascular distribution in reactive nodes and follicular lymphoma while no reproducible vascular pattern is present in diffuse lymphomas. The authors raised the question as to what this means (Hyjek *et al*, 1999) but no explanation has yet been proposed.

The present study was aimed at answering these questions by assessing both the MVD and the vascular phenotype of follicular lymphoma, diffuse large B-cell lymphoma (as defined in the WHO classification) (Jaffe *et al*, 2001) and reactive lymphoid tissues (lymph node and tonsil). We wanted to compare MVD separately within the follicles (reactive and neoplastic) and in the paracortex and to match the MVD data with the vascular phenotype. The aim was to provide a more detailed evaluation of pathways of angiogenesis in the neoplastic and physiological conditions, which would be relevant to antivascular therapy strategies.

To distinguish between mature and immature vessels, two markers were investigated. The first marker was the expression of the LH39 epitope interacting with the antibody LH39, which is expressed on the basal membrane of capillaries and small venules in a variety of normal human tissues but is absent in small vessels present in pyogenic granulomas or nonspecific oral ulceration (Almeida *et al*, 1992a,1992b). These data have suggested that the detection of LH39 staining discriminates between mature and immature vessels, as supported by four further studies on oral carcinomas (Almeida *et al*, 1992a), breast carcinomas (Kakolyris *et al*, 1999) and lung cancer (Kakolyris *et al*, 1999; Passalidou *et al*, 2002).

The second marker examined is integrin *α*V*β*3 that has been reported to be expressed on newly formed endothelium (Brooks *et al*, 1994). Many authors have reported, on brain, skin, kidney and lung, that endothelial cells express it to varying degrees (Rabb *et al*, 1996; Navratil *et al*, 1997; Pazouki *et al*, 1997; Passalidou *et al*, 2002) although in one study of normal human breast tissue the endothelium was negative for *α*V*β*3 (Gasparini *et al*, 1998). While it is well established that endothelial expression of *α*V*β*3 is essential for angiogenesis induced by basic fibroblastic growth factor or tumour necrosis factor (Brooks *et al*, 1994; Friedlander *et al*, 1995), it has also been found that it can be upregulated in resting endothelium by a variety of biological stimuli (Tang *et al*, 1994) and that it is also present on resting endothelial cells (Conforti *et al*, 1992). Study of this integrin remains important as its expression identifies patients who could be eligible for clinical trials with the humanized anti-*α*V*β*3 antibody Vitaxin (Gutheil *et al*, 2000).

## MATERIALS AND METHODS

### Tissue samples

Frozen tissue samples were obtained from the Departments of Histopathology at University College London and at the John Radcliffe Hospital, Oxford. Samples were from 23 patients with follicular non-Hodgkin's lymphoma (of which five had focal transformation to large cell lymphoma), 21 patients with large B-cell non-Hodgkin's lymphoma and 17 with reactive hyperplasia 12 lymph nodes and five tonsils. Fresh tissue was collected in the theatre and frozen in liquid nitrogen. The diagnosis was performed on routine formalin-fixed paraffin-embedded material with the support of appropriate immunostainings according to the WHO classification (Jaffe *et al*, 2001). One section for each staining was evaluated for each patient for microvessels count. The section contained usually all the lymph nodes.

### Immunocytochemistry

The following antibodies were used: the anti-CD34 monoclonal antibody QB10 (DAKO) staining endothelial cells , the antilamina lucida antigen LH39 antibody and the anti-*α*V*β*3 antibody LM609 (Chemicon International, U.K.). Staining for CD3 and CD20 was performed as well. We chose CD34 as endothelial marker as it does not cross-react with lymphoid cells on frozen tissue, as CD31, and stains a wider range of intratumour vessels than anti-Factor VIII antibodies (Vermeulen *et al*, 1995).

All immunostainings were performed on frozen tissue sections. In the single immunostainings, the primary antibody was incubated for 1 h at room temperature. Labelling was performed with an avidin–biotin peroxidase system (DAKO Duet). The appropriate secondary antibody was applied for 35 min, after which the Dako streptavidin–biotin complex was applied followed by DAB solution to develop the staining reaction.

The double immunostaining was performed as follows using the anti-CD34 and anti-LH39 antibodies. Firstly, the anti-LH39 antibody was incubated for 1 h at room temperature. Labelling was performed with an avidin–biotin peroxidase labelling system (DAKO Duet): the appropriate secondary antibody was applied for 35 min, after which the Dako streptavidin–biotin complex was applied and the staining was developed by applying DAB solution. After rinsing, the sections were incubated overnight with the anti-CD34. After further rinsing sections were incubated with rabbit-anti-mouse immunoglobulins and, finally, with APAAP complexes.

All the cells showing staining were scored as positive, independent of the staining intensity. Staining without the primary antibody was routinely carried out as negative control.

### Evaluation of vascularity

In the present study, microvessel is defined, according to the classic definition of Weidner (1995) as ‘capillaries and venules’. We regarded as a distinct countable microvessel any highlighted endothelial cell or cell cluster clearly separated from adjacent microvessels, tumour cells, normal cells and other connective tissue elements according to the international consensus (Vermeulen *et al*, 1996).

In reactive tissue and in follicular lymphomas, the evaluation of vascularity was performed separately for the follicles (either reactive or neoplastic) and for the paracortical area. In follicular lymphoma cases in which focal large cell lymphoma transformation was present (five cases), the area with a diffuse pattern was scored separately. In diffuse large B-cell lymphomas only the scoring of the neoplastic component was performed.

#### Evaluation of MVD

We counted on each section the five vascular hot spots (i.e. the five areas with the higher number of vessels). Having put the vascular hot-spot area under a high-power field (×400 objective), all the vessels present in the field were counted and the value of the sum of the vessels counted in the five spots was calculated.

#### Evaluation of vascular phenotype

To evaluate the proportion of mature vessels, at least 200 vessels were counted from each slide on which double immunostaining for CD34 and LH39 had been performed. If less than 200 vessels were present in the section, they were all counted. The vascular maturation index (VMI) defined by Kakolyris *et al* (1999) as the percentage of LH39/CD34-positive vessels to the total of CD34-positive vessels was calculated.

Similarly, an index was derived comparing vessels positive for *α*V*β*3 to all the CD34-positive vessels. In this case, the staining was performed on serial sections and the count for CD34 on a section was related to the count for *α*V*β*3 on the same areas on the following section. Also in this case, we counted at least 200 CD34-positive vessels from each biopsy. If less than 200 vessels were present, they were all counted. In this case, the index indicates the percent of the vessels expressing *α*V*β*3.

#### Evaluation of vascular regression

Vessels were scored as regressing according to the criteria described by Holash *et al* (1999) on rat experimental tumours: briefly, they performed double immunostaining for Smooth Muscle Actin and for Rat Endothelial Cell Antigen. They classified a vessel as regressing when firstly there was endothelial cell detachment followed by endothelial and smooth muscle cell fragmentation. Their criteria were strictly followed (if in any doubt, the vessels were not scored as regressing). The only difference is that instead of looking at smooth muscle cells the vascular basal membrane was looked at and identified by the expression of LH39 while we used CD34 as human endothelial marker.

The number of regressing vessels was counted compared with the total number of vessels. All the vessels were scored within the follicles present on each section (either reactive or neoplastic) and the vessels present in five randomly chosen high-power fields in the interfollicular areas (reactive tissue and follicular lymphoma cases) or in the areas of diffuse lymphoma.

### Statistical analysis

Comparisons of MVD counts, number of mature vessels (LH39+) and putative immature vessels (*α*V*β*3+) and comparison of regressing vessels, between follicles and paracortex in reactive hyperplasia and follicular lymphomas, were carried out using paired sample *t*-tests since all vessel values were referring to the same sample. There were 17 reactive samples from nodes and tonsils, and 23 follicular lymphomas. All comparisons gave a significant *P*-value at the 95% significance level (Table 1

**Table 1:** Comparison of MVD, number of mature vessels (LH39+) and putative immature vessels (*α*V*β*3+) between follicles and paracortex in reactive hyperplasia and follicular lymphomas (paired samples *t*-test)

	**Follicles**		**Paracortex**	***P*-value**
Reactive, nodes and tonsils (17)				
MVD	68.7	*vs*	282.5	<0.0009
LH39+vessels	77.8	*vs*	97.7	<0.0009
*α*V*β*3+vessels	10.2	*vs*	22.6	=0.044
Follicular lymphoma, All (23)				
MVD	65.9	*vs*	184.6	<0.0009
LH39+vessels	75.8	*vs*	90.4	<0.0009
*α*V*β*3+vessels	45.8	*vs*	66.1	=0.006

and

**Table 4:** Comparison of percentage of regressing vessels between reactive lymphoid tissue, follicular lymphomas and diffuse large B-cell lymphomas (independent samples *t*-test)

	**Follicles reactive**		**Follicles follicular NHL**	***P*-value**	**Paracortex reactive**		**Paracortex follicular NHL**	***P*-value**
Regressing vessels (%)	10.4	*vs*	10.1	=0.940	2.1	*vs*	0.2	=0.223
								
	**Diffuse NHL**		**Follicles reactive**	***P*-value**	**Paracortex reactive**		**Diffuse B NHL**	***P*-value B**
Regressing vessels (%)	0.4	*vs*	10.4	=0.001	2.1	*vs*	0.46	=0.286
								
	**Diffuse NHL**		**Follicles follicular NHL**	***P*-value**	**Diffuse B NHL**		**Paracortex follicular NHL**	***P*-value**
Regressing vessels (%)	0.4	*vs*	10.1	<0.0009	0.4	*vs*	0.2	=0.469

). Independent sample *t*-tests were used for similar comparisons between reactive lymphoid tissue, follicular lymphomas and diffuse large B-cell lymphomas. The *P*-values are given in Table 2

**Table 2:** Comparison of MVD, number of mature vessels (LH39+) and putative immature vessels (*α*V*β*3+) between reactive lymphoid tissue, follicular lymphomas and diffuse large B-cell lymphomas (independent samples *t*-test)

	**Follicles reactive**		**Follicles follicular NHL**	***P*-value**	**Paracortex reactive**		**Paracortex follicular NHL**	***P*-value**
MVD	68.7	*vs*	65.9	=0.785	282.5	*vs*	184.6	<0.0009
LH39+vessels	77.8	*vs*	75.8	=0.689	97.7	*vs*	90.4	=0.007
*α*V*β*3+vessels	10.2	*vs*	45.8	<0.0009	22.6	*vs*	66.1	<0.0009
								
	**Diffuse B NHL**		**Follicles reactive**	***P*-value**	**Diffuse B NHL**		**Paracortex reactive**	***P*-value**
MVD	185.7	*vs*	68.7	<0.0009	185.7	*vs*	282.5	=0.001
LH39+vessels	50.8	*vs*	77.8	=0.007	50.8	*vs*	97.7	<0.0009
*α*V*β*3+vessels	62.7	*vs*	10.2	<0.0009	62.7	*vs*	22.6	<0.0009
								
	**Diffuse NHL**		**Follicles follicular NHL**	***P*-value**	**Diffuse B NHL**		**Paracortex follicular NHL**	***P*-value**
MVD	185.7	*vs*	65.9	<0.0009	185.7	*vs*	184.6	=0.956
LH39+vessels	50.8	*vs*	75.8	=0.007	50.8	*vs*	90.4	<0.0009
*α*V*β*3+vessels	62.7	*vs*	45.8	=0.019	62.7	*vs*	66.1	=0.637

and Table 4.

## RESULTS

### Microvessel density (MVD)

Results of MVD scoring are shown in Table 1 and Table 2. MVD was lower in the follicles, both reactive (germinal centres) and neoplastic follicles, than in the paracortex (Table 1). There was no difference between the MVD in reactive follicles (germinal centres) and neoplastic follicles or between paracortical areas in reactive tissue and in follicular lymphomas (Table 2). MVD in diffuse large cell lymphomas was higher than MVD in the neoplastic follicles and reactive follicles, but lower than the MVD of the paracortex of reactive lymphoid tissues. MVD instead was similar in diffuse large cell lymphomas and in the paracortex of follicular lymphomas (Table 2).

In five cases of follicular lymphoma, an area of diffuse transformation was present. The average score for MVD in these areas was 110, broadly similar to that observed in the diffuse large B-cell lymphomas. Owing to the small number of cases (only five) no statistical analysis was attempted.

### LH39 staining

In reactive lymphoid tissues the staining with LH39 not only demonstrates the presence of positivity in the basal membrane of vessels and lymphatics, but also highlights an LH39 meshwork present throughout the tissue. Both vessels and lymphatics are within this meshwork. Regression of this meshwork is present in the germinal centres. In lymph nodes the meshwork terminates at the capsule while in tonsil, where the lymphatic tissue is extranodal, no discrete boundaries are present.

**Figure 1:**
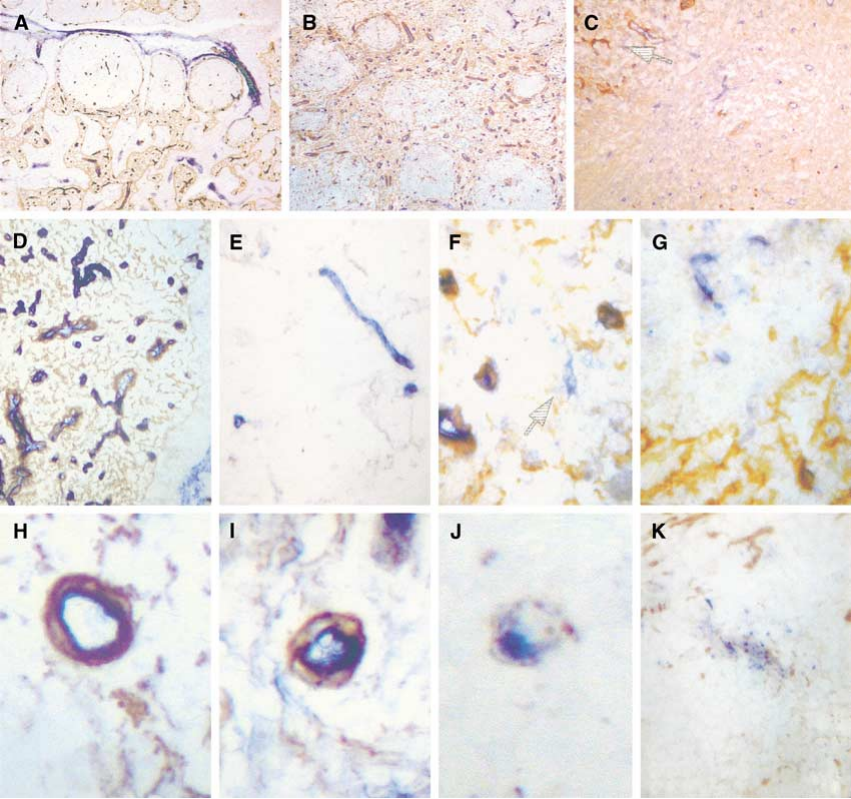
Vascular phenotype in reactive and neoplastic lymph nodes. LH39-positive meshwork in (**A**) a reactive lymph node (**B**) a follicular lymphoma and (**C**) a large B-cell lymphoma; in the latter only a few residual fragments of the LH39 meshwork are present (arrow). (**D**) Reactive lymph nodes: all the vessels in the paracortex are within the LH39 meshwork while (**E**) LH39-negative vessels are present within a germinal centre. (**F**) Only rare LH39-negative vessels are seen in the paracortex in Follicular lymphomas (arrow) while (**G**) some more are present in the neoplastic follicles (arrows). Sequence of vascular regression: (**H**) A normal vessel in which the endothelial cells and the LH39-positive basal membrane are superimposed. (**I**) early detachment of the endothelial cells from the basal membrane. (**J,K**) Progressive fragmentation of both endothelial cells and basal membrane leading to vascular regression.

In follicular lymphomas the LH39 meshwork is similar to that seen in reactive tissue. As in reactive germinal centres, regression of this meshwork is seen within the neoplastic follicles (Figure 1A, B

). The LH39 meshwork is also not present in both large B-cell lymphomas and the focal large cell lymphoma transformations seen in five follicular lymphomas (Figure 1C).

### Vascular phenotype

In Table 1 and Table 2, the data concerning the vascular phenotype in reactive lymphoid tissue (tonsil and lymph node) and lymphomas are summarised.

In the paracortex of the reactive lymph nodes, all the vessels were LH39 positive and were associated with the LH39 meshwork (Figure 1D): LH39-negative vessels could not be detected. In reactive tonsils some LH39-negative vessels were present on the edge of the lymphoid tissue and they accounted for 7.8% of the paracortical vessels. The paracortex of follicular lymphoma nodes differed from the paracortex of reactive nodes as some LH39-negative vessels were seen in the former (Figure 1F). Also, the number of *α*V*β*3-positive vessels was different as less *α*V*β*3-positive vessels were seen in the paracortex of the reactive tissue (Table 2).

Reactive follicles contained instead some LH39-negative vessels (Figure 1E) and the same results were found in neoplastic follicles (Figure 1G). Both in reactive and neoplastic follicles the proportion of mature LH39-positive vessels was the same (Table 2). However, the number of vessels positive for *α*V*β*3 was lower in reactive than in neoplastic follicles.

The proportion of LH39-positive vessels within the large cell lymphomas was lower than that observed in reactive tissue and follicular lymphoma (both in follicles and paracortex). The number of *α*V*β*3 vessels was instead comparable to that seen in follicular lymphomas (follicles and paracortex) and was higher than the number of *α*V*β*3-positive vessels seen in reactive tissue (follicles and paracortex) (Table 2).

### Vascular regression

Results of the scoring for vascular regression are shown in Table 3

**Table 3:** Comparison of percentage of regressing vessels between follicles and paracortex in reactive hyperplasia and follicular lymphomas (paired samples *t*-test)

	**Follicles % regressing vessels**		**Paracortex % regressing vessels**	***P*-value**
Reactive, nodes only (11)	9.0	*vs*	0.2	=0.021
Reactive, nodes and tonsils (16)	10.4	*vs*	2.1^a^	=0.010
Follicular NHL all (21)	10.1	*vs*	0.2	<0.0009

a The high value reported in the paracortex of reactive tonsils is because of a single case of reactive tonsil in which 24% of the paracortical vessels showed signs of regression.

and Table 4. Examples of the vessels scored as regressing are shown in Figure 1H–K. The percentage of regressing vessels was higher in follicles (reactive and neoplastic) than in the paracortex (Table 3). Table 4 shows that the same high proportion of regressing vessels was present in reactive and neoplastic germinal centres, while only occasional regressing vessels were present, in a similar fashion, in large cell lymphomas and in paracortical areas.

## DISCUSSION

The discrepancies in previous studies (Veiga *et al*, 1998; Hyjek *et al*, 1999; Ribatti *et al*, 1999; Vacca *et al*, 1999b; Arias and Soares, 2000; Ridell and Norrby, 2001) concerning MVD values in reactive lymphoid tissues and lymphomas were the primary issue for this investigation. Our data confirm that both in reactive lymph nodes and in lymph nodes with follicular lymphomas, the MVD is higher in the paracortex than in the follicles and that there is no difference in MVD between reactive germinal centres and neoplastic follicles. Finally, it was shown that MVD in the paracortex in reactive nodes is higher than in the paracortex of follicular lymphomas and is also higher than in diffuse large cell lymphomas. This is of interest because of the general consensus that angiogenesis is higher in cancer than in non-neoplastic tissues. However, it is reasonable to think that in the reactive nodes physiologic angiogenesis is more effective than the tumour-induced one.

Therefore, we did not confirm the data suggesting that MVD in lymphomas is higher than in reactive lymphoid tissue (Ribatti *et al*, 1996; Vacca *et al*, 1999b).

The follicular component of follicular lymphomas has an MVD lower than large B-cell lymphomas. However, a number of neoplastic cells in follicular lymphomas are also present in the paracortex where the MVD is comparable to that of large cell lymphomas, suggesting that MVD alone is unlikely to be dictating the aggressiveness of the lymphoma. This finding also suggests that the neoplastic cells colonizing the paracortex might exploit the existing vessels in the same way that we and others (Miliaras *et al*, 1995; Guidi *et al*, 2000; Pezzella *et al*, 2000; Naresh *et al*, 2001) have suggested for solid tumors in reactive nodes.

Our study also revealed a previously unknown anatomical characteristic of the lymph node, that is, an extracellular meshwork identified by the expression of LH39. Within this meshwork, in reactive node, are contained all the vessels. When a reactive germinal centre develops, lysis of this meshwork and vascular regression occurs. This meshwork is also present in extranodal lymphatic tissue, that is, tonsil. As this organ has no discrete boundaries (i.e. a capsule) newly formed LH39 vessels are observed at the edge, probably recruited from the surrounding stroma.

The observation of vascular regression in the germinal centre, associated with neoangiogenesis is the second novel finding. Active vascular remodelling and angiogenesis in adult normal tissues were until now thought to be limited to endometrium, ovary and breast, under the control of cyclic hormonal changes (Augustin, 2000). The occurrence of physiological vascular regression and neoangiogenesis in germinal centres makes it a valuable place to study the pathways regulating normal vascular remodelling (e.g. the angiopoietin pathway) (Holash *et al*, 1999). Furthermore, it also suggests that this could be one of the mechanisms of growth control that alongside others, like the lack of bcl-2, limits the expansion of germinal centre cells. It needs also to be clarified how the vascular regression is regulated and whether apoptosis plays a role in it.

This situation described in reactive nodes is maintained in follicular lymphomas as summarised from the literature (Stewart *et al*, 2002) (Doussis-Anagnostopoulou *et al*, 1997) in Table 5

**Table 5:** Summary of angiogenesis-related features in lymph nodes (data from the present paper, Stewart *et al* (2002) and Doussis-Anagnostopoulou *et al* (1997))

(A) Reactive germinal centres, neoplastic follicles and diffuse large cell NHL			
**NHL**	**Reactive germinal centres**	**Neoplastic follicles**	**Diffuse large B cell**
MVD	69.7	63	**185**
Mature LH39%+	79.6%	76.5%	**50%**
LH39 meshwork	Lysis	Lysis	Lysis
*α*V*β*3+	6.3%	**43%**	**62.7%**
Regressing vessels	9%	10.6%	**0.4%**
Hif1a	+21/25 (84%)	+17/30(56%)	37/48(77%)
Hif2a	Mac	Mac	Mac++
CA IX nuclear	Mac (several)	Mac (several)	**Mac (few)**
CA IX membrane	Absent	Absent	**7 out of 48 (15%)**
VEGF	Mac	**Neoplastic cells**	**Neoplastic cells**
TP cells	Mac/FDR	Mac/FDR	Dendritic like cells
			
(B) Paracortex from reactive lymph nodes, paracortex in lymph nodes with follicular lymphoma and diffuse large cell NHL			
**NHL**	**Reactive LN paracortex**	**FL lymph node paracortex**	**Diffuse large B cell**
MVD	287	**180**	**185**
Mature LH39%+	100%	**90.95%**	**50%**
LH39 meshwork	Present	Present	**Lysis**
*α*V*β*3+	20.7%	**66%**	**62.7%**
Regressing vessels	0.2%	0.2%	0.4%
Hif1a	Rare	Rare	37 out of 48 cases (77%)
Hif2a	Several	Several	mac++
CA IX nuclear	Absent	Absent	**Mac (few)**
CA IX membrane	Absent	Absent	**7 out of 48 cases (15%)**
VEGF	Mac few	**Neoplastic cells**	**Neoplastic cells**
TP	Mac/IDR	Mac/IDR	Dendritic-like

with only three differences: a higher expression of *α*V*β*3, the presence in the paracortex of a few immature (LH39-negative) vessels and the expression of VEGF on the neoplastic cells. The observations by Hyjek *et al* (1999) that mostly mature vessels are present in follicular lymphomas and that the same overall vascular distribution is seen in follicular lymphoma and reactive nodes could be explained by the fact that a similar angiogenic pathway might be activated in both of these conditions. However, our data showing a difference, small but significant, in LH39-negative and *α*V*β*3-positive vessels suggest that in the paracortex of follicular lymphomas a second angiogenic pathway is appearing.

A different situation is found in large B-cell lymphomas: regression of the LH39 meshwork occurs while only occasional regressing vessels are seen. This finding supports the hypothesis that in follicular lymphoma the neoplastic cells retain the ability to balance neoangiogenesis with vascular regression. This characteristic is lost in large cell lymphoma and could be another factor contributing to their aggressiveness.

Secondly, although large cell lymphomas have a lower MVD than the reactive paracortex, the phenotype of the vessels is different. Half of them are immature (LH39-negative) and *α*V*β*3 is widely expressed. These data indicate that the angiogenic pathway producing immature vessels becomes predominant during the progression from follicular to diffuse lymphoma. Furthermore, the balance between neoangiogenesis and vascular regression, still maintained in neoplastic follicles, is lost in diffuse large cell lymphomas. Large B-cell lymphomas therefore have a vascular phenotype comparable to that observed in solid tumours (Kakolyris *et al*, 1999,^2000^; Passalidou *et al*, 2002).

Lymphatic vessels have not been investigated in this study. Efforts have been made to exclude them on morphological examination but the recent identification of the molecule Lyve 1 and the raising of a specific antibody against this protein (Skobe *et al*, 2001), which is specifically expressed on lymphatic vessels, will allow this to be properly investigated in normal and neoplastic lymph nodes in future studies.

The heterogeneous patterns of vascularity observed in this study suggest that different types of approach may be needed to exploit anti-angiogenic treatment in lymphomas. In follicular lymphomas, where vessels are mostly mature but with *α*V*β*3 expression, the humanised anti-*α*V*β*3 antibody Vitaxin (Gutheil *et al*, 2000) might be suitable. In contrast, antiangiogenic drugs interacting with the formation of new vessels may be more effective in diffuse large B-cell lymphomas.

In conclusion, our data support the hypothesis that follicular lymphomas grow in a fashion very similar to that of reactive lymph nodes while large B-cell lymphomas have angiogenic patterns similar to that seen in many epithelial tumors.
